# Metabolic Surgery to Treat Obesity in Diabetic Kidney Disease, Chronic Kidney Disease, and End-Stage Kidney Disease; What Are the Unanswered Questions?

**DOI:** 10.3389/fendo.2020.00289

**Published:** 2020-08-17

**Authors:** William P. Martin, James White, Francisco J. López-Hernández, Neil G. Docherty, Carel W. le Roux

**Affiliations:** ^1^Diabetes Complications Research Centre, School of Medicine, Conway Institute of Biomolecular and Biomedical Research, University College Dublin, Dublin, Ireland; ^2^Instituto de Estudios de Ciencias de la Salud de Castilla y León-Instituto de Investigación Biomédica de Salamanca (IECSCYL-IBSAL), Hospital Virgen Vega, Salamanca, Spain; ^3^Division of Investigative Science, Imperial College London, London, United Kingdom

**Keywords:** obesity, metabolic surgery, type 2 diabetes mellitus, diabetic kidney disease, chronic kidney disease, end-stage kidney disease, kidney transplantation, dialysis

## Abstract

Obesity is a major factor in contemporary clinical practice in nephrology. Obesity accelerates the progression of both diabetic and non-diabetic chronic kidney disease and, in renal transplantation, both recipient and donor obesity increase the risk of allograft complications. Obesity is thus a major driver of renal disease progression and a barrier to deceased and living donor kidney transplantation. Large observational studies have highlighted that metabolic surgery reduces the incidence of albuminuria, slows chronic kidney disease progression, and reduces the incidence of end-stage kidney disease over extended follow-up in people with and without type 2 diabetes. The surgical treatment of obesity and its metabolic sequelae has therefore the potential to improve management of diabetic and non-diabetic chronic kidney disease and aid in the slowing of renal decline toward end-stage kidney disease. In the context of patients with end-stage kidney disease, although complications of metabolic surgery are higher, absolute event rates are low and it remains a safe intervention in this population. Pre-transplant metabolic surgery increases access to kidney transplantation in people with obesity and end-stage kidney disease. Metabolic surgery also improves management of metabolic complications post-kidney transplantation, including new-onset diabetes. Procedure selection may be critical to mitigate the risks of oxalate nephropathy and disruption to immunosuppressant pharmacokinetics. Metabolic surgery may also have a role in the treatment of donor obesity, which could increase the living kidney donor pool with potential downstream impact on kidney paired exchange programmes. The present paper provides a comprehensive coverage of the literature concerning renal outcomes in clinical studies of metabolic surgery and integrates findings from relevant mechanistic pre-clinical studies. In so doing the key unanswered questions for the field are brought to the fore for discussion.

## Obesity Is a Disease

Clinicians understand the risks of being obese and encourage patients to lose weight to prevent the complications of obesity. Thus, far, it has been very difficult to treat obesity as the aetiology has been misunderstood. Logically, if clinicians can convince themselves that obesity is a disease that requires treatment then progress can be made. Most human diseases are characterised by a set of reproducible symptoms and signs which affect a particular group of people and follow a predictable clinical trajectory (1). Our understanding of disease is enhanced when its aetiology and complications are well defined. Obesity can now be defined as a disease characterised by the pathognomonic symptoms of excess hunger and/or reduced satiation after a meal and the pathognomonic sign of increased adiposity, resulting in a state of dysregulated energy homeostasis that substantially increases mortality (2). Subcortical brain regions, especially the hypothalamus, integrate a diverse array of endocrine, neural, and neuroendocrine afferent signals from the viscera and external cues to govern human feeding behaviours (3). Evidence has been mounting that will help clinicians understand that by approaching obesity as a disease of the subcortical brain regions, more effective and compassionate treatments can be provided to our patients.

## Diabetic Kidney Disease and Obesity-Related Glomerulopathy: an Overview of the 2 Major Drivers of Chronic Kidney Disease in People With Obesity

### Diabetic Kidney Disease (DKD)

The obesity pandemic has contributed to an increased prevalence of type 2 diabetes and its attendant microvascular complications, including diabetic kidney disease (DKD) (4). DKD develops in ~40% of people with type 2 diabetes, with higher prevalence among non-Caucasian ethnicities (5). DKD is associated with significant increases in cardiovascular and all-cause mortality, with the majority of excess cardiovascular and all-cause mortality attributable to diabetes occurring in those with kidney disease (6). Compared with people with diabetes but no kidney disease, DKD [defined as eGFR ≤60 mL/min/BSA and/or urine albumin-to-creatinine ratio (uACR) ≥3 mg/mmol] increased the absolute risk of 10-year all-cause mortality by 19.6% in the Third National Health and Nutrition Examination Survey (6). The pathophysiology of DKD is primarily driven by metabolic dysregulation accompanying diabetes (hyperglycaemia, dyslipidaemia, and hypertension), and clinical management of DKD focuses on control of these inciting factors through blood pressure control, renin-angiotensin-aldosterone system (RAAS) blockade, and intensification of glycaemic and lipid control to minimise proteinuria and slow eGFR decline (7). Two recent additions to the DKD treatment paradigm include sodium-glucose co-transporter-2 inhibitors (SGLT2is) and GLP-1 receptor analogues (GLP1RAs), which independently of improvements in glycaemic control and blood pressure, have been demonstrated to improve renal outcomes in people with type 2 diabetes (8, 9). Obesity is now recognized as a risk factor for the onset and progression of type 2 DKD (7), yet obesity treatments are not currently offered to people with type 2 DKD in routine clinical practice.

Pathophysiological alterations which perpetuate DKD can be considered separately as haemodynamic (RAAS activation, endothelial dysfunction), metabolic (accumulation of advanced glycation end-products), pro-inflammatory (reactive oxygen species generation, tumour necrosis factor-α activation), and pro-fibrotic (stimulation of transforming growth factor-β signaling) (7), but in reality these elements interact locally and systemically to form a complex and dynamic interplay resulting in functional and structural changes to the kidney (10). Structural kidney changes are more heterogeneous in type 2 compared with type 1 DKD and include arteriolar hyalinosis, glomerular and tubular basement membrane thickening, mesangial matrix expansion, and podocyte foot process effacement (11). In later stages, extreme mesangial expansion may lead to nodular glomerulosclerosis and formation of pathognomonic Kimmelstiel-Wilson lesions (11). Tubular atrophy and interstitial fibrosis also occur late in the disease process (11).

A diagnosis of DKD is assigned in the presence of persistent albuminuria (uACR ≥3 mg/mmol) and/or sustained reduction in eGFR ≤60 mL/min/BSA in patients with suggestive clinical features, including presence of other diabetes microvascular complications (e.g., diabetic retinopathy), negative autoimmune serology, and absence of an active urinary sediment (e.g., dysmorphic red blood cells or red blood cell casts) (7). The natural history of type 2 DKD remains incompletely elucidated. Previously, most patients were considered to progress sequentially through phases including glomerular hyperfiltration, microalbuminuria, and macroalbuminuria with progressive eGFR decline (12). It is now recognized that a significant number of people with DKD experience progressive eGFR decline in the absence of albuminuria (13), highlighting the need for biomarkers which facilitate earlier diagnosis and prognostication of cardiovascular disease and end-stage kidney disease (ESKD) risk among people with DKD.

### Obesity-Related Glomerulopathy (ORG)

The obesity pandemic has also fueled an increased incidence of obesity-related glomerulopathy (ORG), a distinct cause of chronic kidney disease (CKD) characterized by sub-nephrotic range proteinuria, glomerulomegaly, and progressive renal functional loss (14). Histological evidence of glomerulomegaly is the defining feature of ORG and is present in all biopsy-proven cases (14). In the absence of confirmation by kidney biopsy, and where other kidney diseases are not suspected based on features such as accelerated renal functional decline or an active urinary sediment, persistent proteinuria in obese patients without diabetes is generally considered diagnostic of ORG (14). Obesity increases afferent arteriolar ultrafiltration pressure to cause glomerular hyperfiltration (15); as a result, many patients with ORG also develop an adaptive form of focal segmental glomerulosclerosis (FSGS) (16). FSGS is defined as segmental solidification of the glomerular tuft by extracellular matrix and/or hyaline, with obliteration of capillaries in sclerosed segments (16). Six forms (primary, adaptive, APOL1-associated, high-penetrance genetic, virus-associated, medication-associated) and five histologic subtypes (perihilar, cellular, tip, collapsing, not otherwise specified) of FSGS are recognized (16). FSGS complicating ORG is considered an adaptive response to a mismatch between glomerular load and capacity, has a predilection for perihilar sclerosis, and generally affects fewer glomeruli and results in less podocyte foot process effacement than primary FSGS (14). As a result, adaptive FSGS accompanying ORG rarely causes full-blown nephrotic syndrome and runs a more indolent course than primary FSGS, although renal survival at 10 years from diagnosis is only 50% (17).

Similar to DKD, multiple pathophysiological disruptions, both systemically and locally in the kidney, provoke and perpetuate ORG. Indeed, many parallels exist between DKD and ORG pathogenesis, including glomerular hyperfiltration, RAAS overactivation, enhanced tubular sodium reabsorption, hypoadiponectinaemia, renal lipotoxicitiy, and impaired tubular fatty acid β-oxidation. The potential of metabolic surgery to reverse many of these phenomena simultaneously [discussed further below in the section Mechanisms Underpinning Renoprotection and in other recent reviews from our group (18)] supports a growing interest in the potential utility of metabolic surgery to treat both DKD and ORG. Glomerular hyperfiltration in ORG occurs via afferent arteriolar dilatation, resulting in abnormal transmission of increased arterial pressure to glomerular capillaries in individuals with systemic hypertension (15). Enhanced proximal tubular sodium reabsorption occurs in people with obesity, which in turn decreases distal tubular sodium delivery to the macula densa, diminishes tubuloglomerular feedback, and leads to compensatory afferent arteriolar vasodilatation to result in glomerular hyperfiltration (19). Adipose tissue synthesizes RAAS components, and obese individuals demonstrate RAAS overactivation (20). Increased leptin and decreased adiponectin synthesis by adipose tissue in obese individuals also activates the renal sympathetic nervous system (RSNS) (21). RAAS and RSNS overactivation, along with hyperinsulinaemia, further perpetuate tubular sodium reabsorption, glomerular hyperfiltration, and systemic hypertension in obese individuals (14). Obesity-associated hypoadiponectinaemia may worsen proteinuria through a direct action on podocytes (22). Beyond haemodynamic alterations, expansion of specific visceral adipose tissue depots including peri-renal fat and renal sinus fat may mechanically compress the kidney in obese individuals, while renal lipotoxicity arising from renal triglyceride and cholesterol accumulation may also exacerbate ORG (23).

The true prevalence of ORG is unknown, although the prevalence of significant proteinuria (≥1+ by urine dipstick or uACR ≥30 mg/mmol) is 4–10% amongst obese patients (24, 25). Importantly, over 45% of patients with histologically confirmed ORG in a single-centre study in the United States had a BMI <40 kg/m^2^ (26). Therefore, ORG is not restricted to those with extreme BMIs and should be highly prevalent in those attending routine weight management services. Furthermore, in a study of 95 patients with BMI ≥40 kg/m^2^ without overt kidney disease (normal serum creatinine and <0.2 grams proteinuria/day) who underwent kidney biopsy at the time of metabolic surgery, a wide range of histological glomerular abnormalities were present including glomerulomegaly, mesangial matrix expansion, podocyte hypertrophy, and glomerulosclerosis (27). Therefore, subclinical kidney disease with established glomerular structural abnormalities is likely severely under-recognised in patients attending weight management services. Non-invasive serum and urinary biomarkers which accurately identify such patients with subclinical ORG are urgently needed. RAAS blockade, which reduces efferent arteriolar resistance to lower glomerular hydrostatic pressure, is the main approach to ORG management (16). Dietary sodium restriction, thiazide diuretics, and aldosterone antagonists may be used to potentiate the anti-proteinuric effects of RAAS blockade, although such regimens require further study (16). Prospective, controlled studies of metabolic surgery in individuals with biopsy-proven ORG and proteinuric CKD are required.

## The Obesity Pandemic Is Contributing to Increasing Chronic Kidney Disease and End-Stage Kidney Disease Prevalence: Evidence From Observational Studies and Systematic Reviews and Meta-Analyses

Obesity complications are well defined and their rising prevalence presents one of the foremost challenges to healthcare delivery in the 21st century. Importantly, many complications including hypertension (28), type 2 diabetes (29), obstructive sleep apnoea (30), idiopathic intracranial hypertension (31), polycystic ovarian syndrome (32), and non-alcoholic fatty liver disease (33) can be reversed with intentional weight loss. Compared with other obesity complications, comparatively little is known about kidney disease as a complication of obesity. The increasing prevalence of obesity, CKD, and ESKD are inter-related (34). Obesity increases risk of CKD onset and of progression of existing CKD to ESKD (34).

Chang et al. performed a meta-analysis of individual participant data to investigate relationships between adiposity measures (body-mass index, waist circumference, and waist-to-height ratio), glomerular filtration rate (GFR) decline, and all-cause mortality (35). Over mean 8-year follow-up in *n* = 39 general population cohorts (*n* = 5,459,014 participants), hazard ratios for BMIs 30, 35, and 40 were 1.18 (95% CI 1.09–1.27), 1.69 (95% CI 1.51–1.89), and 2.02 (95% CI 1.80–2.27), respectively, for increased risk of GFR decline compared with BMI of 25. The impact of BMI on subsequent GFR decline in people with established CKD was smaller. Across 18 CKD cohorts (*n* = 91,607 participants) over mean follow-up of 4 years, the hazard ratio for GFR decline for BMI 35 vs. 25 was 1.17 (95% CI 1.04–1.31). Similar to BMI, increasing waist circumference and waist-to-height ratio were strongly associated with GFR decline in general population cohorts and more weakly associated with GFR decline in CKD cohorts. Increasing BMI and waist circumference, but not waist-to-height ratio, were also associated mortality risk in people with CKD. Independent of relationships between obesity and type 2 diabetes, this data provides a rationale to pursue dedicated obesity treatments to reduce CKD prevalence and progression.

Similarly, Garofalo et al. performed a systematic review and meta-analysis to investigate relationships between BMI and both impaired eGFR (<60 mL/min/BSA) and/or albuminuria (1+ on urinary dipstick or uACR ≥3.4 mg/mmol) (36). Across 39 cohorts (*n* = 630,677 participants) with mean follow-up of 6.8 years, obesity but not overweight increased the relative risk of onset of low eGFR (1.28, 95% CI 1.07–1.54) and albuminuria (1.51, 95% CI 1.36–1.67). Each unit increase in BMI (kg/m^2^) increased the relative risk of onset of both eGFR <60 mL/min/BSA and albuminuria by 2%.

Multiple epidemiologic studies have also demonstrated that obesity strongly and independently increases the risk of ESKD. Hsu et al. demonstrated that increasing severity of obesity incrementally increased the risk of ESKD compared with controls with normal BMI (18.5–24.9 kg/m^2^) in *n* = 320,252 adults with and without baseline CKD (37). Adjusted relative risks for ESKD were 1.87 (95% CI 1.64–2.14), 3.57 (95% CI 3.05–4.18), 6.12 (95% CI 4.97–7.54), and 7.07 (95% CI 5.37–9.31) for individuals with overweight, class 1 obesity, class 2 obesity, and class 3 obesity, respectively. Similarly, Lu et al. demonstrated that BMI ≥35 kg/m^2^ independently increased risk of adverse renal outcomes (steeper eGFR slope, doubling of serum creatinine, and incident ESKD) in *n* = 453,946 United States veterans with baseline CKD (eGFR <60 mL/min/BSA), although associations weakened in those with more advanced CKD (38).

## Metabolic Surgery Rearranges the Gut to Effectively Treat Obesity

**Figure 1:**
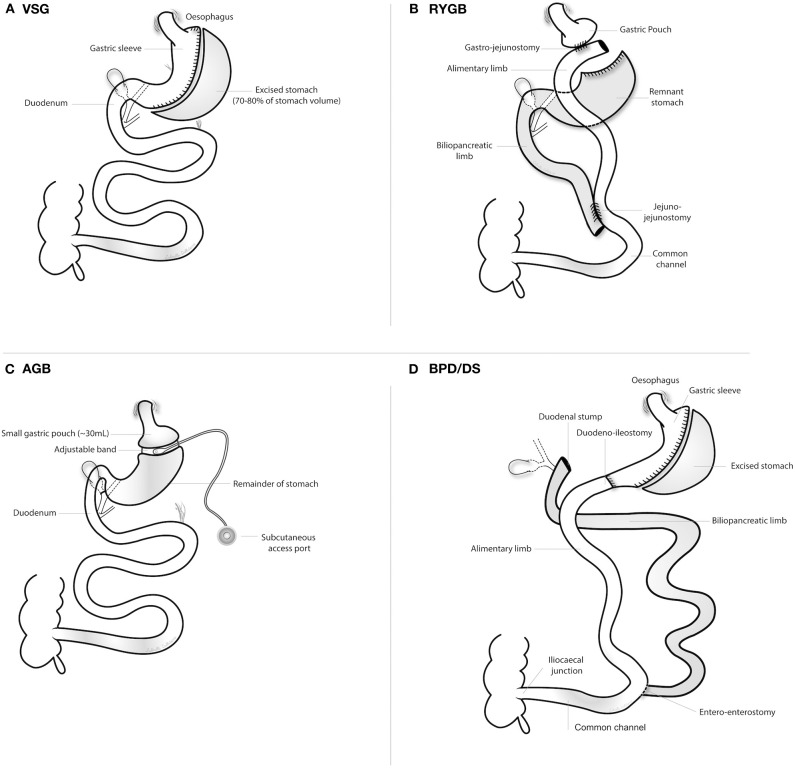
Overview of the four principal metabolic surgery types. AGB, adjustable gastric banding; BPD/DS, biliopancreatic diversion with duodenal switch; RYGB, Roux-en-Y gastric bypass surgery; VSG, vertical sleeve gastrectomy. **(A)** vertical sleeve gastrectomy; **(B)** Roux-en-Y gastric bypass surgery; **(C)** adjustable gastric banding; **(D)** biliopancreatic diversion with duodenal switch.

Figure 1 outlines the four main metabolic surgery types discussed in the context of CKD and ESKD management in this review: vertical sleeve gastrectomy (VSG), Roux-en-Y gastric bypass (RYGB), adjustable gastric banding (AGB), and biliopancreatic diversion/duodenal switch (BPD/DS). All procedures can be performed laparoscopically (39). Description of the rearranged gastrointestinal anatomy for each of the four procedures is summarized below:

- VSG (Figure 1A) (39)
  – The stomach is excised (70–80%) along the greater curvature. The remnant tubular stomach contains the lesser curvature that is resistant to stretching due to the absence of the fundus, creating a high-pressure chamber that causes rapid gastric emptying and early delivery of ingested food to the small intestine.
- RYGB (Figure 1B) (39, 40):
  – The stomach is transected to create a small gastric pouch (15–30 mL volume) and a gastric remnant excluded from direct contact with food.
  – A jejunotomy is performed in the mid-jejunum, 50–75 cm distal to the ligament of Treitz.
  – An alimentary/Roux limb (100–150 cm length) is created by anastomosing the gastric pouch to the distal limb of the jejunotomy, allowing ingested food to bypass the duodenum and proximal jejunum and directly enter the mid-jejunum.
  – The small intestine proximal to the jejunotomy forms the biliopancreatic limb, which transports secretions from the remnant stomach, biliary tree, and pancreas.
  – The common channel is formed by side-to-side anastomosis of the biliopancreatic and alimentary limbs 100–150 cm distal to the gastrojejunostomy, and major absorption and digestion of nutrients occurs downstream of this juncture.
  – An alimentary limb of length 150 cm does not increase the risk of nutritional complications but does improve metabolic control compared with shorter limb lengths (41); alimentary limb lengths up to 150 cm are now the standard form of RYGB employed in clinical practice. Modification of the length of the alimentary limb beyond 150 cm (“long-limb RYGB”) may be used to achieve even greater weight loss and metabolic control, although the risk for nutritional deficiencies, enteric hyperoxaluria, and suboptimal absorption of immunosuppressive agents also increases (42).
- AGB (Figure 1C) (39):
  – A silicone ring (band) is inserted around the stomach, immediately below the gastro- oesophageal junction, to create a 30 mL upper gastric pouch and a narrow passage to the remaining stomach.
  – The band is connected to a subcutaneous infusion port, wherein the volume of saline can be adjusted to control the band diameter.
- BPD/DS (Figure 1D) (39):
  – A VSG is combined with a proximal duodenoileostomy, causing ingested food to rapidly enter the small intestine and then bypass the duodenum and jejunum.

AGB has declined in popularity, accounting for 5–10% of metabolic surgeries performed in the United States, due to modest efficacy and high complication rates requiring band revisions and/or removal (43). In the 2018 IFSO Global Bariatric Surgery Registry Report, which amalgamated data from 51 countries, AGB accounted for 5.0% of all metabolic surgeries performed since 2014 (44). BPD/DS is now infrequently performed due to the high burden of postoperative nutritional deficiencies resulting from the more extensive intestinal bypass (45). VSG and RYGB are the 2 most commonly performed metabolic surgeries. VSG has supplanted RYGB as the most commonly performed metabolic surgery procedure worldwide over the past decade (43, 46). Nevertheless, RYGB remains the most commonly performed metabolic surgery in South America and comprises ~40% of metabolic surgeries performed in the United States (43, 47). Compared with VSG, RYGB induces superior weight loss and metabolic control (29), and a much larger body of evidence with extended follow-up supports its efficacy in controlling diabetes complications (48). Nevertheless, VSG has dramatically increased in popularity as it is a relatively simple procedure with lower operative morbidity, and reduces the risks of internal herniation and micronutrient deficiency. At 1-year post-metabolic surgery in people with obesity and type 2 diabetes, RYGB reduced mean body weight by 4.3 kg more than VSG (49). Although further head-to-head comparisons between RYGB and VSG should be conducted in a randomised fashion, it appears that weight loss after VSG ranges between that of RYGB and AGB (50). Beyond anticipated metabolic benefits, other individual patient characteristics influence choice of metabolic surgery procedure. For example, gastro-oesophageal reflux disease (GORD) is common in obese individuals (51). *De novo* GORD and aggravation of pre-existing GORD is more common after VSG than RYGB (52), and given the relationship between obesity, GORD, and increasing incidence of oesophageal adenocarcinoma (53), RYGB may be preferable in this setting. Indeed, several large observational studies have highlighted that proton-pump inhibitors increase risk for acute interstitial nephritis and CKD onset and progression (54–56); therefore, minimising proton-pump inhibitor exposure after metabolic surgery by favouring RYGB in those at high-risk for *de novo* or aggravated GORD postoperatively may also be beneficial for kidney health.

Metabolic surgery achieves sustained weight loss and substantially lowers mortality in people with obesity (57, 58). Obesity is increasingly recognised as an important driver of CKD progression and is an important barrier to kidney transplantation, yet obesity is not specifically addressed during routine management of CKD and ESKD despite evidence of benefit (59). We herein summarise evidence supporting the potentially diverse roles of metabolic surgery in slowing DKD and CKD progression, as well as facilitating access to kidney transplantation and management of post-transplant metabolic complications. The primary purpose of the current review is to critically review clinical studies investigating the role of metabolic surgery in the management of patients with kidney disease of all stages. A limited discussion of mechanisms underpinning renoprotection conferred by metabolic surgery is provided in the section Mechanisms Underpinning Renoprotection. We refer the reader interested in mechanisms of renoprotection after metabolic surgery to a related recent review from our group (18).

## What Is the Role of Metabolic Surgery in the Management of Type 2 Diabetic Kidney Disease?

### RCTs

Evidence supporting a role of metabolic surgery in the management of type 2 DKD is currently derived from RCTs of metabolic surgery for type 2 diabetes control, and observational studies investigating changes in renal parameters after metabolic surgery in people with obesity and type 2 diabetes. Table 1 provides an overview of the major metabolic surgery RCTs which have investigated type 2 diabetes control and remission in obese individuals (29, 49, 60–67). Partial and complete diabetes remission were defined as HbA_1c_ < 6.5% and <6.0% without hypoglycaemic medication usage, respectively, although Dixon et al. defined diabetes remission as HbA_1c_ < 6.2% and fasting plasma glucose <126 mg/dL (67). All studies used intensive medical intervention as a control arm. In the largest head-to-head study (STAMPEDE RCT), RYGB achieved superior weight loss at 5 years compared with VSG (29). Additionally, complete and partial type 2 diabetes remission rates were higher in the RYGB arm compared with the VSG arm at 5 years (29). BPD/DS resulted in higher rates of partial and complete diabetes remission at 5 years than RYGB, despite similar weight loss in both groups (65).

**Table 1:** Anthropometry, type 2 diabetes control, and albuminuria in metabolic surgery RCTs in patients with obesity and type 2 diabetes.

**Study**	**Study arms**	**Follow-up (months)**	**Body weight (kg)**	**BMI (kg/m^**2**^)**	**T2D duration (years)**	**HbA_**1*c***_ (%)**	**HbA_**1*c***_ ≤6.5% without diabetes medications [*n* (%)]**	**HbA_**1*c***_ ≤6% without diabetes medications [*n* (%)]**	**Albuminuria^a^ [*n* (%)]**
**Schauer et al. (STAMPEDE)** **(****29**, **49**, **60****)**									
	**RYGB** **+** **IMT**	0	107 ± 15	37 ± 3	8 ± 6	9 ± 1	–	–	17 (34)
		12	77 ± 13	27 ± 3	–	6 ± 1	–	21 (42)	–
		36	81 ± 16	28	–	7 ± 1	22 (46)	17 (35)	–
		60	83 ± 15	29	–	7 ± 2	15 (31)	11 (22)	9 (19)
	**VSG** **+** **IMT**	0	101 ± 16	36 ± 4	9 ± 5	10 ± 2	–	–	12 (24)
		12	76 ± 13	27 ± 4	–	7 ± 1	–	13 (27)	–
		36	79 ± 15	29	–	7 ± 1	14 (29)	10 (20)	–
		60	82 ± 15	29	–	7 ± 2	11 (23)	7 (15)	5 (11)
	**IMT**	0	107 ± 15	37 ± 3	9 ± 6	9 ± 1	–	–	10 (20)
		12	99 ± 16	34 ± 4	–	8 ± 2	–	0 (0)	–
		36	100 ± 17	35	–	8 ± 2	0 (0)	0 (0)	–
		60	99 ± 17	34	–	9 ± 2	0 (0)	0 (0)	8 (22)
**Ikramuddin et al. (DSS)** **(****61****–****63****)**									
	**RYGB + IMT**	0	99 ± 14	35 (34-36)	9 ± 6	10 ± 1	–	–	–
		12	73 ± 14	26 (25-27)	–	6 ± 1	0 (0)	0 (0)	–
		24	–	27 (26-27)	–	7 ± 2	25 (42)	15 (25)	–
		60	–	27 (27-28)	–	7 (7-8)	9 (16)	4 (7)	–
	**IMT**	0	98 ± 17	34 (34-35)	9 ± 6	10 ± 1	–	–	–
		12	90 ± 17	32 (31-32)	–	8 ± 2	0 (0)	0 (0)	–
		24	–	32 (31-33)	–	8 ± 3	0 (0)	0 (0)	–
		60	–	31 (30-32)	–	9 (8-9)	0 (0)	0 (0)	–
**Mingrone et al**. **(****64**, **65****)**									
	**RYGB + IMT**	0	130 ± 23	45 ± 5	6 ± 1	9 ± 1	–	–	3 (16)
		24	84 ± 13	29 ± 3	–	6 ± 1	15 (75)	–	–
		60	90 ± 13	31 ± 3	–	7 ± 1	8 (42)	1 (5)	0 (0)
	**BPD/DS + IMT**	0	138 ± 30	45 ± 8	6 ± 1	9 ± 2	–	–	2 (11)
		24	90 ± 18	29 ± 5	–	5 ± 0.5	19 (95)	–	–
		60	93 ± 14	30 ± 4	–	6 ± 0.4	13 (68)	7 (37)	0 (0)
	**IMT**	0	136 ± 22	46 ± 6	6 ± 1	9 ± 1	–	–	4 (27)
		24	128 ± 20	43 ± 6	–	8 ± 1	0 (0)	0 (0)	–
		60	127 ± 21	42 ± 6	–	7 ± 1	0 (0)	0 (0)	4 (27)
**Cummings et al. (CROSSROADS) (****66****)**									
	**RYGB + IMT**	0	109 ± 15	38 ± 4	11 ± 5	8 ± 1	–	–	–
		12	–	–	–	6 ± 2	9 (60)	9 (60)	–
	**ILMI**	0	113 ± 17	37 ± 4	7 ± 5	7 ± 1	–	–	–
		12	–	–	–	7 ± 1	1 (6)	1 (6)	–
**Dixon et al**. **(****67****)**									
	**AGB + IMT**	0	106 ± 14	37 ± 3	–	8 ± 1	–	–	–
		24	85 ± 16	–	–	6 ± 1	–	22 (73)^b^	–
	**IMT**	0	106 ± 14	37 ± 3	–	8 ± 1	–	–	–
		24	105 ± 15	–	–	7 ± 1	0 (0)	4 (13)^b^	–

a *Albuminuria, urine albumin/creatinine ratio ≥3 mg albumin per mmol of creatinine*.

b *Type 2 diabetes remission defined as HbA_1c_ <6.2% and fasting plasma glucose <126 mg/dL without hypoglycaemic medication usage in this study*.

*Mean ± SD or mean (95% CI) are presented for normally distributed continuous variables; n (%) are presented for categorical variables*.

*AGB, adjustable gastric banding; BMI, body-mass index; BPD/DS, biliopancreatic diversion/duodenal switch; DSS, Diabetes Surgery Study; HbA1_c_, glycated haemoglobin; ILMI, intensive lifestyle and medical intervention; IMT, intensive medical therapy; RYGB, Roux-en-Y gastric bypass; T2D, type 2 diabetes mellitus; VSG, vertical sleeve gastrectomy*.

Despite the early diabetes-remitting effects of metabolic surgery, high rates of type 2 diabetes recurrence over longer follow-up are now recognized. In the STAMPEDE RCT, of the 20 patients that achieved complete diabetes remission at 1 year post-RYGB, 10/20 (50%) experienced diabetes relapse by 5 years (29). Similarly, of the 12 patients that achieved complete diabetes remission at 1 year post-VSG, 5/12 (41.7%) experienced diabetes relapse by 5 years (29). Miras et al. randomised people with persistent or recurrent type 2 diabetes after metabolic surgery (HbA_1c_ > 6.5% at 1 year post-RYGB or VSG) to liraglutide 1.8 mg once daily or placebo, combined with a reduced-calorie diet and increased physical activity (68). Liraglutide reduced HbA_1c_ by 1.2% and body weight by 4.2 kg compared with the placebo arm at 26 weeks, with no difference in treatment response by surgery type. Thus, liraglutide may be used to optimize metabolic outcomes after metabolic surgery. Semaglutide achieved even greater reductions in body weight and glycaemia than liraglutide in the SUSTAIN clinical trial programme (69), and could also be potentially used to improve type 2 diabetes control after metabolic surgery. Liraglutide reduced DKD onset and progression in the LEADER RCT (9); thus, its use post-metabolic surgery may also confer additional renoprotective benefits. While questions remain as to the optimal type 2 diabetes medication algorithm post-metabolic surgery, it is clear that combined surgical and medical treatment approaches offer the most intensive control and should be used to optimize renal outcomes in those with type 2 DKD.

Two RCTs of metabolic surgery for type 2 diabetes reported changes in albuminuria postoperatively (29, 65) (Table 1). Although numbers with baseline albuminuria were small in Mingrone et al.'s RCT (*n* = 3 RYGB, *n* = 2 BPD/DS), both surgeries achieved sustained remission of albuminuria in all patients at 5 years (65). Prevalence of baseline albuminuria was higher in the STAMPEDE RCT, with RYGB reducing the proportion of patients with albuminuria from *n* = 17 (34%) at baseline to *n* = 9 (19%) at 5 years (29). Similarly, VSG reduced albuminuria prevalence from *n* = 12 (24%) at baseline to *n* = 5 (11.1%) at 5 years. Despite the low numbers of patients with albuminuria recruited to these studies, such data highlights the potent albuminuria-lowering effect of metabolic surgery. No RCTs have yet assessed the impact of metabolic surgery on primary renal outcomes in people with type 2 DKD. The Microvascular Outcomes after Metabolic Surgery (MOMS) clinical trial, which randomised people with obesity (BMI 30–34.9 kg/m^2^), type 2 diabetes and moderately increased albuminuria to best medical care plus Roux-en-Y gastric bypass surgery or best medical care alone, finished recruiting in 2016; 1, 2, and 5 year outcomes are awaited (40). Individuals with baseline eGFR <30 mL/min/BSA were excluded from the MOMS RCT and baseline eGFR was >60 mL/min/BSA in both study arms (40). An RCT which randomizes individuals with obesity, type 2 diabetes, severely increased albuminuria, and eGFR 30–60 mL/min/BSA to metabolic surgery plus best medical care or best medical care alone and assesses mGFR in a longitudinal fashion is required to determine the impact of metabolic surgery in those with more advanced type 2 DKD.

### Observational Studies

#### Incidence of DKD

Several large scale-observational studies have examined the impact of metabolic surgery on subsequent incidence of microvascular complications of diabetes, including type 2 DKD. O'Brien et al. performed a retrospective observational cohort study of 4,024 people with type 2 diabetes who underwent metabolic surgery between 2005 and 2011 across one of four health care systems in the United States, and 11,059 matched non-surgically treated controls (70). Metabolic surgery reduced incident nephropathy (defined as sustained CKD-EPI eGFR <60 mL/min/BSA over ≥90 days) at 7-year follow-up, with kidney disease developing in 6.4% [95% CI 5.3–7.6%] of surgically treated patients and 14% [95% CI 13–15.1%] of non-surgical controls (adjusted hazard ratio 0.45 [95% CI 0.29–0.71]).

Madsen et al. performed a matched cohort study to examine the impact of RYGB on type 2 diabetes remission and micro- and macro-vascular complications of diabetes in an obese (BMI ≥35 kg/m^2^), Northern Danish population (71). RYGB-operated subjects (*n* = 1,111) and were compared to 1,074 matched controls. Type 2 diabetes remission occurred in 74% of RYGB-operated individuals at 1 year postoperatively; however, 27% of these relapsed at 5-year follow-up. RYGB reduced the risk of microvascular complications of type 2 diabetes over 5.3 year follow-up (HR 0.53 [95% CI 0.38–0.73]). Similarly, reduction in incident microvascular complications was greater in those that achieved type 2 diabetes remission at 1-year follow-up compared with those that did not (HR 0.43 [95% CI 0.25–0.72]). RYGB reduced the development of type 2 DKD compared with matched controls (incidence rate ratio 0.54 [95% CI 0.31–0.94]).

A subgroup analysis of people with baseline type 2 diabetes in the Swedish Obese Subjects (SOS) cohort study also identified reduced incidence of microvascular complications of diabetes after metabolic surgery (*n* = 343) compared with controls who received usual care (*n* = 260) (hazard ratio 0.44 [95% CI 0.34–0.56]) (48). Although the impact of metabolic surgery on renal endpoints is not specifically reported in this paper, a subsequent publication from the SOS study highlighted that metabolic surgery (*n* = 187) reduced the subsequent incidence of albuminuria in those with baseline type 2 diabetes compared with controls (*n* = 157) over median 10-year follow-up (hazard ratio 0.35 [95% CI 0.21–0.59]) (72). Similarly, Shulman et al. demonstrated that metabolic surgery (*n* = 344) in the SOS cohort reduced the incidence of a composite of CKD stage 4/ESKD in those with baseline type 2 diabetes compared with usual care controls (*n* = 263) over median 18-year follow-up (hazard ratio 0.35 [95% CI 0.15–0.81]) (73). Numbers needed to treat with metabolic surgery to prevent one case of albuminuria and CKD stage 4/ESKD were 4 and 46, respectively, in those with baseline type 2 diabetes (72, 73). Considering that DKD often progresses to ESKD which is costly to manage (74, 75), metabolic surgery for the prevention of ESKD is likely cost-effective although formal cost-effectiveness analysis is required.

#### Remission/Control of Existing DKD

Heneghan et al. investigated changes in albuminuria 5 years after metabolic surgery in 52 people with type 2 diabetes at a single centre (*n* = 36 RYGB, *n* = 13 AGB, *n* = 3 VSG) (76). Of those with baseline albuminuria (31.3 and 6.3% with moderately and severely increased albuminuria, respectively), 58.3% achieved remission of albuminuria at 5-year follow-up. Conversely, moderately increased albuminuria developed over 5 years in only 25% of those without baseline albuminuria, while no patients developed severely increased albuminuria during follow-up. The anti-albuminuric effect of metabolic surgery associated with postoperative improvement or remission of type 2 diabetes.

Canney et al. investigated the impact of RYGB in addition to optimal medical therapy in 105 individuals with type 2 diabetes (mean duration 13 ± 3 years) and moderately or severely increased albuminuria (77). At mean 13-month follow-up, mean BMI reduced from 38 to 27 kg/m^2^, mean HbA_1c_ decreased from 8 to 6%, and median uACR decreased by 81% from 88 to 14 mg/g. Remission of albuminuria (uACR <30 mg/g) occurred in 82 (78%) people postoperatively. Reductions in albuminuria were seen across all tertiles of baseline albuminuria indicating that RYGB is an effective anti-proteinuric strategy across the spectrum of baseline DKD severity. A parallel pre-clinical study of RYGB in the Zucker Diabetic Fatty rat model of obesity and DKD identified similar reductions in albuminuria postoperatively (mean 86% reduction in uACR) (77). Moreover, RYGB reduced glomerular volume, podocyte stress (desmin expression), and podocyte dedifferentiation (podocyte foot process effacement assessed by transmission electron microscopy) indicating that the anti-proteinuric effect of RYGB is accompanied by concordant improvements in glomerular structure and ultrastructure.

### Future Directions

Thus, several RCTs indicate that metabolic surgery combined with medical therapy significantly improves type 2 diabetes control while multiple, large observational studies indicate that such metabolic benefits result in improved renal outcomes over the longer term. With recent advances in medical care of diabetes, including SGLT2is and GLP1RAs, opportunities to optimise metabolic parameters and end-organ damage in the kidney abound through combined metabolic surgery plus intensive medical care. Metabolic surgery will not serve as a sole and primary treatment modality for DKD, but rather serve as adjunct to optimal medical therapy to halt progressive DKD. Definitive RCTs investigating the impact of metabolic surgery in people with type 2 diabetes, impaired glomerular filtration, and progressive renal functional decline despite optimal medical therapy are necessary to define its role across the spectrum of CKD severity.

## What Is the Role of Metabolic Surgery for Slowing the Progression of Chronic Kidney Disease?

Although metabolic surgery dramatically improves diabetes control in many patients, its renoprotective effects occur independently of improvements in glycaemia and BMI (78). Scheurlen et al. performed a systematic review and meta-analysis of changes in albuminuria after metabolic surgery in people with type 2 diabetes, and their correlation with changes in HbA_1c_, BMI, and systolic blood pressure (79). The authors identified 15 suitable studies of mixed study design (five retrospective cohort studies, eight prospective cohort studies, one prospective case-control study, and one randomised controlled trial). No relationship between change in albuminuria and changes in BMI, HbA_1c_, and systolic blood pressure after metabolic surgery were identified. Such observations emphasise the importance of pre-clinical studies of metabolic surgery with direct access to kidney tissue, wherein weight-independent and glycaemia-independent mediators of renoprotection can be interrogated.

An increasing body of observational evidence indicates that metabolic surgery also improves renal outcomes in people without diabetes. The major observational studies are summarized below. No RCTs have yet investigated the impact of metabolic surgery in those with non-diabetic CKD. Future prospective studies should ideally be conducted in a randomized fashion, using 24-h urinary protein excretion rates and measured glomerular filtration rate as primary outcomes, to investigate renoprotective effects of metabolic surgery in this setting.

### Observational Studies

#### Matched Observational Cohort Studies (No Baseline CKD)

Chang et al. investigated the impact of metabolic surgery on renal outcomes in 985 people (baseline type 2 diabetes in 37.8%; RYGB 96.5%, VSG 3.5%) and an equal number of matched controls (80). The surgery and control cohorts were followed for a median of 4.4 and 3.8 years, respectively. Metabolic surgery reduced the risk of ≥30% eGFR decline by 58% (hazard ratio 0.42 [95% CI 0.32–0.55]). Similarly, metabolic surgery also lowered a composite endpoint of doubling of serum creatinine or ESKD by 57% (hazard ratio 0.43 [95% CI 0.26–0.71]).

Risk reductions in renal outcomes of similar magnitude were also reported in the SOS study, in which the baseline type 2 diabetes prevalence was significantly smaller and in which a much lower proportion of patients underwent RYGB. Carlsson et al. investigated the incidence of albuminuria over median 10-year follow-up in 1,498 surgically treated patients (baseline diabetes in 12.5%; gastric banding 18%, vertical banded gastroplasty 69%, RYGB 13%) and 1,610 matched controls (72). Metabolic surgery reduced the incidence of albuminuria by 63% (adjusted hazard ratio 0.37 [95% CI 0.30–0.47]), with the greatest reductions seen in those who received RYGB (69% reduction in albuminuria incidence). Shulman et al. highlighted that metabolic surgery reduced the incidence of ESKD after median 18-year follow-up in the SOS study by over 70% (adjusted hazard ratio 0.27 [95% CI 0.12–0.60]) (73). No matched observational cohort studies of metabolic surgery have selectively recruited people with CKD at baseline.

#### Observational Studies in Those With Baseline CKD

Friedman et al. investigated the impact of metabolic surgery (RYGB 71%, AGB 24%, remaining VSG, BPD/DS, and banded gastric bypass) on KDIGO CKD risk classification over up to 7 years in the Longitudinal Assessment of Bariatric Surgery (LABS)-2 cohort (81). At baseline, the majority of people were at low CKD risk (1,788, 83%), while 254 (12%), 73 (3%), and 29 (1%) were at moderate, high, and very high CKD risk, respectively, and by definition had CKD. Diabetes prevalence ranged from 28% in those with low CKD risk to 83% in those with very high CKD risk. CKD risk categories reduced for a majority of people with moderate (53% improved categories) and high (56% improved categories) CKD risk at 7 years postoperatively. Furthermore, 23% of those with very high baseline CKD risk improved their risk category by 7 years, indicating that metabolic surgery continues to have renoprotective benefits, albeit less pronounced, across the spectrum of CKD severity.

Romero-Funes et al. studied changes in CKD-EPI_creatinine_ eGFR, albuminuria, and kidney failure risk before and at 12 months after metabolic surgery in *n* = 69 people (VSG 61%, RYGB 39%; 93% diabetes) at Cleveland Clinic, Florida, United States (82). In *n* = 20 (29%) people with CKD stage 3 or greater, uACR decreased from 66.5 [35.1–465.4] to 47 [25.1–66] mg/g at 12-month follow-up. Similarly, in *n* = 37 (54%) people with moderately or severely increased albuminuria, uACR decreased from 80 [47.5–389.1] to 46 [30–89.7] mg/g at 12 months. In those with CKD stage 3 or greater, these reductions in proteinuria contributed to 70 and 60% relative reductions in estimated risk of kidney failure at 2 and 5 years, respectively. However, these kidney failure risk estimates also included postoperative changes in CKD-EPI_creatinine_ eGFR, which are not reliable in the setting of lean muscle loss post-metabolic surgery (83). Nevertheless, metabolic surgery in people with established CKD effectively reduces proteinuria and subsequent risk of ESKD.

### Minimising the Potential for Enteric Hyperoxaluria

Enteric hyperoxaluria is common post-RYGB but not VSG. Untreated obesity itself increases the risk of kidney stones (84), but stone formation rates increase after metabolic surgery and are higher after RYGB than VSG. Lieske et al. investigated stone formation rates after metabolic surgery (*n* = 762; 78% RYGB, 14% long-limb RYGB or BPD/DS, 7% VSG or AGB) compared with non-surgical controls (*n* = 759) matched by BMI in Olmsted County, Minnesota, United States (85). Baseline CKD and nephrolithiasis prevalence were ~10 and 4% in both groups, respectively. Over mean 6-year follow-up, incident stone events were more common in metabolic surgery patients than obese controls (11 vs. 4%, *p* < 0.01) and a very high proportion of stones post-metabolic surgery were composed of calcium oxalate (94%). Stone risk was highest after long-limb RYGB or BPD/DS, intermediate after standard RYGB, and lowest after VSG or AGB. Amongst individuals who underwent metabolic surgery, those with stones preoperatively were more likely to form further stones postoperatively (42 vs. 14% at 10 years, *p* < 0.001), reflecting the known phenomenon that stone event risk increases with the number of prior events (86). Amongst people who formed stones post-metabolic surgery, urinary oxalate excretion almost doubled, urinary citrate excretion decreased by 30%, and calcium oxalate supersaturation increased by 25% beyond 8 months postoperatively.

Thus, kidney stones are common after metabolic surgery and the increased incidence is driven by hyperoxaluria, hypocitraturia, and consequently urinary calcium oxalate supersaturation (85). Usually, dietary calcium and oxalate bind in the gut lumen, precipitate as calcium oxalate, and are excreted in stool (87). Faecal fat excretion increases after RYGB (88); non-absorbed fatty acids preferentially bind calcium in the gut lumen minimising the amount of dietary calcium available to bind oxalate (87). The concentration of soluble oxalate increases in the colon, where it passively diffuses into the bloodstream. Postoperative increases in luminal fatty acids and bile acids may increase colonic permeability to oxalate (89, 90). Modifying gut microbial flora after RYGB, specifically increasing colonisation with *Oxalobacter formigenes* which protects from hyperoxaluria by degrading luminal oxalate, holds promise as a means of decreasing enteric oxalate absorption (91). Oxalate is filtered by the kidneys and excreted unchanged in the urine. The risk of urinary supersaturation with oxalate may be further increased post-RYGB by suboptimal fluid intake and hypocitraturia (85).

However, enteric hyperoxaluria is eminently controllable after standard RYGB when multi-disciplinary care is delivered with pre- and post-operative dietary education. Reduced dietary oxalate consumption and oral calcium supplementation minimise intestinal oxalate absorption (92). A low-fat diet can also minimise intestinal oxalate absorption (92), although may be nutritionally inadequate for some people postoperatively. Increased fluid intake (reduced supersaturation of stone-forming salts) and oral citrate supplementation in those with hypocitraturia (crystallisation inhibitor) also reduce stone events (93). While long-limb RYGB significantly increases stone events (85), the differential impact of shortening of the alimentary limb in a standard RYGB on metabolic control, stone event rates, and net renal outcomes remains an open question which should be addressed through a prospective, randomised study. A head-to-head comparison of the impact of RYGB with 150 vs. 75 cm alimentary limb length on weight loss, metabolic control, 24-h urinary supersaturation profiles, and mGFR in people with CKD could prove valuable in the refinement of surgical practice for RYGB in patients with extant renal disease in order to maximise benefit. In the meantime, given the high rate of new kidney stone formation post-metabolic surgery in those with baseline nephrolithiasis (85), it may be prudent to favour VSG to minimise hyperoxaluria in this group of patients for now.

### Mechanisms Underpinning Renoprotection

**Figure 2:**
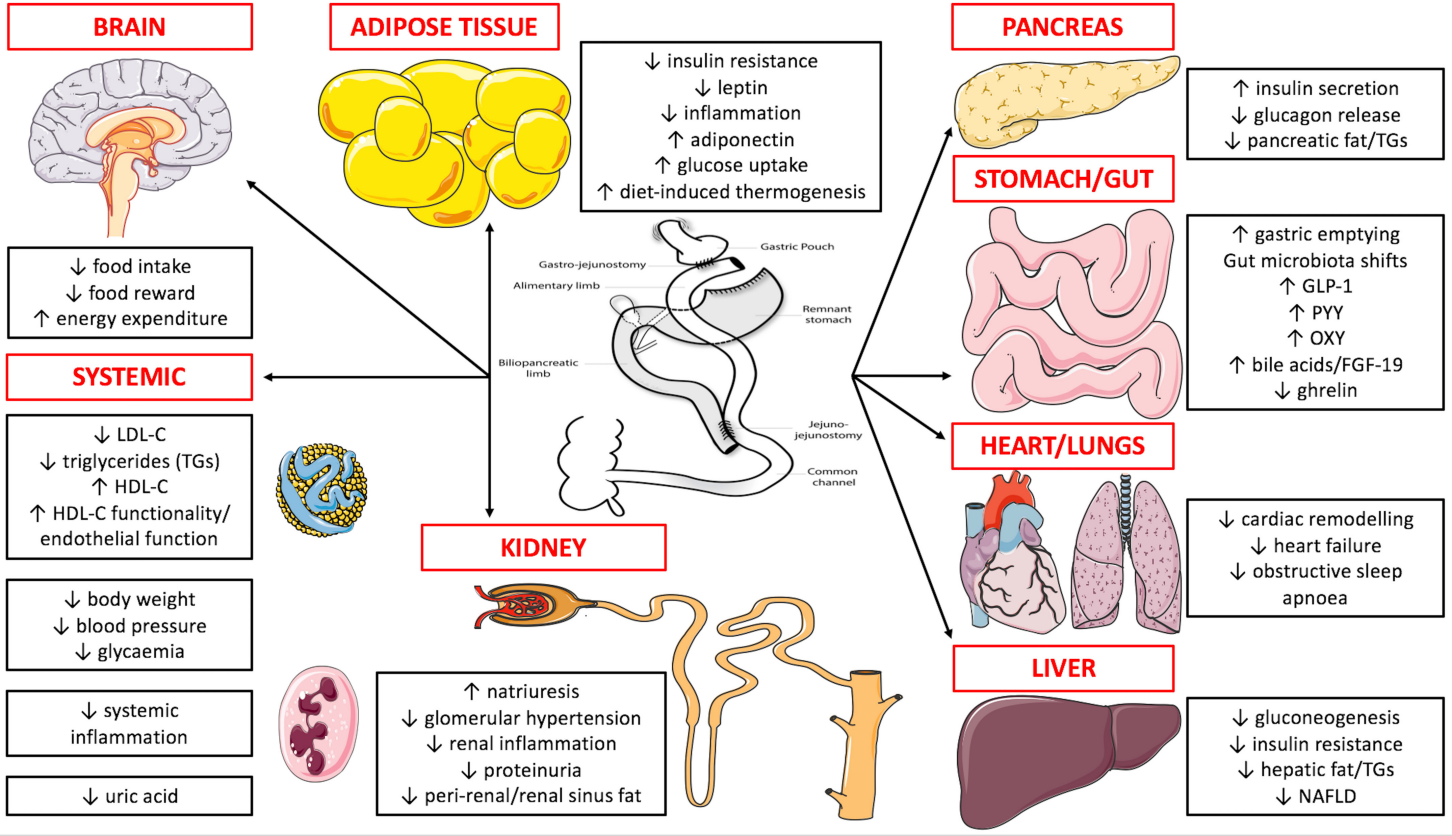
Overview of mechanisms underpinning weight loss, improved glycaemia, and renoprotection after metabolic surgery. RYGB is depicted in the figure as more mechanistic data supports its weight loss, glycaemic, and renoprotective benefits compared with other metabolic surgery procedures. FGF-19, fibroblast growth factor-19; GLP-1, glucagon-like peptide-1; HDL-C, high-density lipoprotein-cholesterol; LDL-C, low-density lipoprotein-cholesterol; NAFLD, non-alcoholic fatty liver disease; OXY, oxyntomodulin; PYY, peptide YY; TGs, triglycerides. **Figure 2** was created by adapting images downloaded from the image bank of Servier Medical Art (https://smart.servier.com/). Servier Medical Art by Servier is licensed under a Creative Commons Attribution 3.0 Unported License (CC BY 3.0: https://creativecommons.org/licenses/by/3.0/).

Figure 2 summarises mechanisms underpinning weight loss, improved glycaemia, and renoprotection after metabolic surgery. We have recently reviewed these mechanisms in detail (18, 94). More recently developed mechanistic insights include reductions in serum uric acid and increases in serum uromodulin post-metabolic surgery (95, 96). It is clear that metabolic surgery exerts diverse effects across multiple organ systems and systemically in a simultaneous fashion to exert renoprotective effects. While it is intuitive to focus on improved metabolic control as the primary driver of renoprotection post-metabolic surgery, renoprotective effects occur independently of these changes and so interrogation of weight-independent mediators is of critical importance (78). Increased glucagon-like peptide-1 post-metabolic surgery may be central to its renoprotective effects, contributing to the metabolic, antihypertensive, anti-inflammatory, and natriuretic benefits of the procedure (94, 97). We herein summarise the pathogenic role of ectopic renal fat accumulation in people with obesity, and the potential contribution of changes in these visceral fat depots after metabolic surgery to its renoprotective effects.

In obesity, ectopic fat accumulation on, around, and within abdominal viscera, may occur and contribute to pathologies (98). The left and right kidneys have a thick and multi-layered covering of adipose tissue. With reference to the kidneys, the para-renal fat lies external to the renal fascia, whilst deep to the renal fascia and external to the renal capsule lies the peri-renal fat which extends into the renal sinus at the hilum wherein it invests the renal vasculature. With increasing abdominal adiposity, increased fat deposition occurs within the renal sinus and surrounding the kidney. Obesity and visceral adiposity are established risk factors for CKD, but specific fat depots such as extra- and intra-renal fat have gained attention recently for their local and systemic contributions to disease (99–101).

With the use of magnetic resonance, computed tomography, and ultrasound imaging modalities, multiple adipose tissue compartments can be quantified (102, 103). With these tools cross-sectional population studies and longitudinal studies have investigated what contribution kidney fat expansion makes to kidney function and CKD development. Renal sinus fat (RSF) is significantly correlated with visceral adipose tissue volume (104). RSF volume is greater in males than females, and the left kidney has a significantly larger volume of RSF than the right kidney in both genders (102). In a large community population from the Framingham Heart Study, 30% of the population were described as having a high presence of RSF, termed “fatty kidney” (104). In this population, fatty kidney was associated with CKD. This association remained significant after adjustment for visceral adipose tissue (VAT) and BMI, indicating RSF may have a contribution to CKD that is independent of other fat compartments. RSF has been correlated with other markers of kidney function such as serum creatinine, serum kidney injury molecule-1 and serum fibroblast growth factor-21 (102, 105). In a cross-sectional exercise study RSF was not associated with eGFR or uACR at baseline, but had a significant association with uACR post-exercise, after adjustment for multiple variables including VAT (106). In a study of people with type 2 diabetes, RSF had an association with mGFR that remained significant after adjustment for BMI and VAT, but not after adjustment for age (107).

Similar to RSF, peri-renal fat volume is also greater in males and on the left side (108). Para-renal and peri-renal ultrasonographic fat thickness (PUFT) was associated with CKD-EPI eGFR, renal resistance index, and serum urate levels in people with type 2 diabetes (109). PUFT predicted these outcomes independently of waist circumference and BMI by multivariable regression analysis, and was a stronger predictor than waist circumference and BMI. Incidence of CKD also increased significantly across tertiles of increasing PUFT (109). In people with CKD, peri-renal fat thickness (PRFT) associates with metabolic risk factors for CKD progression such as higher fasting blood glucose, elevated blood triglycerides and hyperuricemia (110).

Several mechanisms have been suggested to explain ectopic kidney fat's association with metabolic dysfunction and CKD. One mechanism is that ectopic kidney fat exerts a physical force upon the kidney. RSF may compress the renal medulla (98). The renal vein and renal lymph vessels may be compressed by surrounding RSF, leading to decreased flow and an increase in pressure and size of the kidney. Animal studies have reported increased extracellular matrix in the medulla and increased renal interstitial hydrostatic pressure in obesity models (19, 111, 112). Hall et al. describes how this increased pressure compresses the vasa recta and loop of Henle to increase tubular reabsorption of sodium (113). This mechanism is supported by an association in humans between RSF and kidney size that persists after adjustment for age, sex, BMI and height (105).

Peri-renal fat is usually measured on images as the fat pad between the posterior surface of the kidney and the abdominal wall musculature (109, 114). Unlike the RSF, it is separated from the renal parenchyma by the fibrous renal capsule. It still may however contribute toward increasing intra-abdominal pressure and physical compression upon the kidney (115). Peri-renal fat shares the same vascularization as the renal cortex which may permit adipokines and cytokines from the fat depot to have a paracrine effect on the kidney (114). Infiltration of macrophages into peri-renal fat tissue and polarisation of macrophages toward a pro-inflammatory state has been documented in high-fat diet mice (116, 117). A paracrine effect of RSF mediated through locally secreted cytokines and vasoconstrictive factors has also been suggested as a link between RSF and renal function. One study found an association between RSF, mGFR and renal vascular resistance, a parameter implicated in the progression of CKD (107). Spit et al. hypothesize that the increased inflammation and reduced adiponectin from obese adipose tissue may mediate reduced renal vasorelaxation (107). However, when blood markers of inflammation were measured no association with RSF was found, suggesting that peri-renal fat and RSF depots are too small to have independent associations with systemic inflammation (102).

As adipose tissue expands in obesity its ability to store energy may become impaired, causing lipolysis and fatty acid release to occur (118). In their proximity to the kidney, dysfunction in the renal sinus and peri-renal fat depots may lead to lipid deposition and lipotoxicity in the kidney. Parenchymal lipid accumulation in kidney glomeruli and proximal tubules is observed in mice when fed a high-fat, but not a low-fat, diet (119). Zucker diabetic fatty rats, a model of obesity and type 2 diabetes, display intra-renal lipid accumulation (mostly in the proximal tubules) whereas lean control animals display no intra-renal lipid accumulation (120). Intra-glomerular lipid deposition and intracellular accumulation of fat are common in routine human kidney biopsies (121). Increased lipid accumulation and alterations in fatty acid beta-oxidation in proximal tubular cells promotes renal inflammation and fibrogenesis (122). Lipid accumulation in podocytes has been shown to induce insulin resistance and alterations to the actin cytoskeleton (123).

Several longitudinal studies have been performed to investigate what contribution ectopic renal fat reduction has on renal function. In a study where diet-induced weight loss occurred over 18 months, RSF correlated with eGFR and microalbuminuria at baseline (124). RSF decreased by 8.6% after 18 months and correlated with total body weight loss. Decreased RSF was associated with improvements in dyslipidaemia and glycaemic control. Improvements in uACR and uric acid associated with decreased RSF, but the association was no longer significant after adjusting for VAT. In a study of people with severe obesity, PRFT was significantly higher in hypertensive patients than those with normal blood pressure, and was significantly associated with systolic blood pressure (114). At 12 months after VSG, PRFT was significantly decreased (114). In the hypertensive group, those with higher baseline PRFT showed greater reduction in antihypertensive medications. Similarly, RSF has been found to be associated with number of prescribed antihypertensive medications and more severe hypertension (105).

The term “fatty kidney disease” has recently been coined to describe the accumulation of ectopic fat on and within the kidney. It is hoped that designating fatty kidney as a distinct clinical entity will help to focus research efforts (125). In longitudinal studies of changes in renal ectopic fat depots after metabolic surgery, it remains a challenge to separate the effect of RSF reduction from concomitantly occurring VAT and total body fat reductions (107). Despite this, future studies of metabolic surgery should include measurements of renal sinus and peri-renal fats pre- and post-intervention to further characterise the contribution of ectopic kidney fat reduction to its renoprotective effects.

### Future Directions

The impact of metabolic surgery on kidney function and injury extends beyond corrections in metabolic parameters, supporting its potential utility in people with non-diabetic CKD. Obesity-related glomerulopathy is under-recognised yet increasing in prevalence (26). Even in obese people with apparently normal kidney function, distinct renal histopathological lesions including glomerulosclerosis, glomerulomegaly mesangial matrix expansion, and podocyte hypertrophy are highly prevalent, and can potentially be improved with metabolic surgery (27, 126). Furthermore, routine care of people with non-immune-mediated CKD centres on control of blood pressure and proteinuria but does not usually involve obesity treatment (127). Metabolic surgery may serve as an adjunct to intensify control of renal parameters in obese people with persistent renal functional decline and/or proteinuria despite treatment with a maximally tolerated dose of a RAAS inhibitor and/or SGLT2i (if subsequently approved for non-diabetic CKD) (128, 129). Thus, metabolic surgery may have an increasing role in outpatient nephrology care.

Reductions in proteinuria after metabolic surgery occur independently of postoperative improvements in body weight, blood pressure, and glycaemic control, suggesting that metabolic surgery has distinct renoprotective effects beyond correction of conventional risk factors for CKD progression (78). Elucidation of these mechanisms is a priority for successful translation of metabolic surgery to nephrology practice, and also to potentially non-invasively mimic and harness metabolic surgery's renoprotective benefits. The majority of evidence supporting the renoprotective effects of metabolic surgery pertains to RYGB. However, VSG is now the most commonly performed metabolic surgery procedure worldwide (43, 46) and, while adverse renal effects including enteric hyperoxaluria and kidney stones are less common after VSG, it is important to determine if VSG shares all or some of RYGB's renoprotective effects. The impact of modification of the length of the standard RYGB alimentary limb should be investigated to determine if a shorter limb length can achieve comparable metabolic control and weight-independent renoprotective benefits while minimising hyperoxaluria risk to improve net renal outcomes. Additionally, nutritional deficiencies in iron, calcium, and vitamin D after metabolic surgery may exacerbate anaemia and mineral bone disease as complications of CKD (130). Oxalosis as a consequence of enteric hyperoxaluria may also result in erythopoeitin-stimulating agent resistant anaemia (131). Thus, strategies to mitigate nutritional complications of metabolic surgery to minimise adverse impact on anaemia and bone health parameters in people with CKD should be sought.

## What Is the Role of Metabolic Surgery in People With End-Stage Kidney Disease?

Although higher BMI is associated with lower mortality in people with ESKD on maintenance dialysis (132), the so-called obesity paradox, obesity is also a major barrier to kidney transplantation in people with ESKD as recipient obesity increases the risk of kidney allograft complications (133). Almost all kidney transplant centres avoid kidney transplantation in prospective recipients with a BMI ≥40 kg/m^2^, while most centres consider a BMI ≥35 kg/m^2^ a relative contraindication (134). Some centres employ a more stringent BMI cutoff of ≤30 kg/m^2^ for transplant waitlisting (134). Survival and quality of life for people with ESKD on dialysis, particularly with diabetes, is extremely poor (135, 136). Quality of life and survival significantly improve after kidney transplantation, irrespective of BMI (137). Therefore, intentional weight loss strategies hold promise as a means of decreasing mortality rates of people with obesity, type 2 diabetes, and ESKD by increasing successful kidney transplantation listing rates.

### What Evidence Supports Medical Weight Management Options in People With Obesity and End-Stage Kidney Disease?

Experience with medical treatment of obesity in people with ESKD is limited. MacLaughlin et al. investigated the impact of orlistat plus exercise and dietary advice (*n* = 32) vs. usual care (*n* = 20) over 24 months in people with advanced CKD (31.3% type 2 diabetes, 37.5% stage 3/4 CKD, and 62.5% on dialysis in orlistat group) (138). Baseline BMIs were 35.7 ± 4.5 and 34.1 ± 4.2 kg/m^2^ in the orlistat and usual care groups, respectively. At 24 months, body weight significantly decreased in the orlistat group compared with the usual care group (94.6 ± 16.1 vs. 101.0 ± 26.8 kg, *p* < 0.001). Comparing the orlistat and usual care groups, more people were accepted for kidney transplant listing (35 vs. 6%) and were transplanted (3 vs. 1). Predictably, orlistat caused gastrointestinal side effects but these were not severe enough to result in participant withdrawal. No patients developed hyperoxaluria, although this remains a concern given the fat malabsorption caused by orlistat (one of the same mechanisms implicated in the enteric hyperoxaluria accompanying RYGB) (139, 140).

Idorn et al. randomised 20 people with type 2 diabetes and ESKD and 20 control subjects with type 2 diabetes but no kidney disease in a 1:1 fashion to liraglutide or placebo (141). Participants were followed for 12 weeks and liraglutide was titrated to a maximum dose of 1.8 mg once daily as tolerated. Liraglutide improved glycaemic control and reduced insulin requirements in people with and without ESKD. Liraglutide-induced weight loss was greater in people without kidney disease than in those with ESKD, with body weight decreasing by 2.4 ± 0.8 kg in those with ESKD (*p* = 0.22). Plasma trough liraglutide concentrations were increased by 49% in the ESKD group compared with the control group by 12 weeks, and gastrointestinal adverse effects were more pronounced in the ESKD group. Thus, liraglutide appears to retain glycaemic and weight management efficacy in people with ESKD, although tolerability diminishes somewhat and slower titration may be preferable. Liraglutide-induced constipation is common (~20% incidence in phase 3 RCTs of liraglutide in people without kidney disease) (142); no studies have yet investigated the tolerability of liraglutide in people on peritoneal dialysis in whom avoidance of constipation is of paramount importance (143).

### Can Metabolic Surgery Facilitate Access to Kidney Transplantation for People With Dialysis-Dependent End-Stage Kidney Disease?

**Figure 3:**
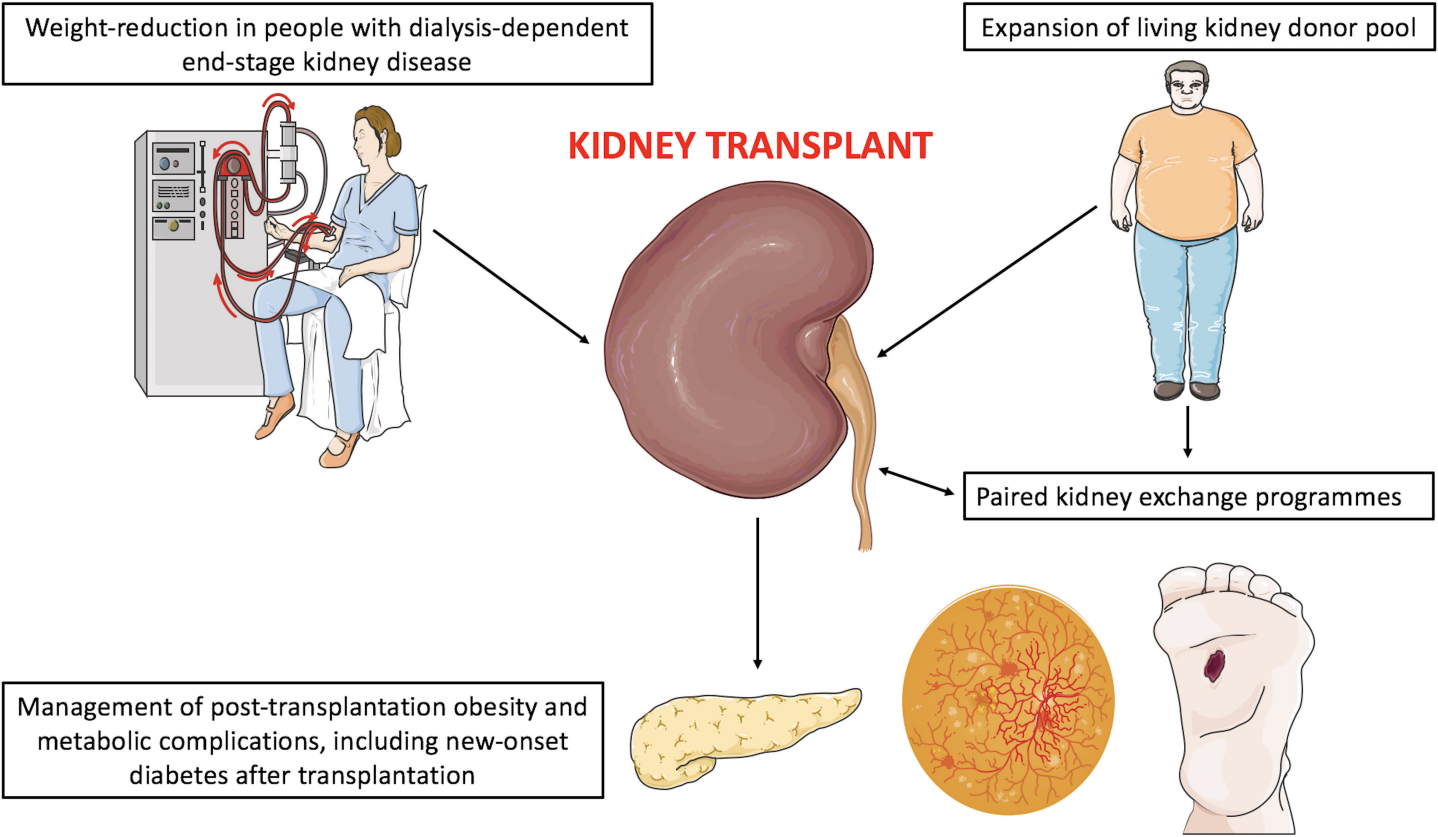
Potential roles of metabolic surgery in facilitating access to kidney transplantation and improving allograft outcomes in people with obesity and end-stage kidney disease. Although not visually represented, pre-transplant laparoscopic metabolic surgery has been successfully performed in people on peritoneal dialysis without necessitating temporary haemodialysis. However, reports to date are limited and further study is warranted to determine surgical and dialysis complication rates in this population. **Figure 3** was created by adapting images downloaded from the image bank of Servier Medical Art (https://smart.servier.com/). Servier Medical Art by Servier is licensed under a Creative Commons Attribution 3.0 Unported License (CC BY 3.0: https://creativecommons.org/licenses/by/3.0/).

Compared with patients without ESKD, metabolic surgery in ESKD does not increase the relative risk of postoperative complications, but does slightly prolong length of hospital stay and increases 30-day hospital readmission rates (144). Absolute complication rates remain relatively low (2.5–6.4%) in people with ESKD, and metabolic surgery appears to be a safe and efficacious option for weight management in ESKD (144). Indeed, Sheetz et al. analysed United States Medicare claims data between 2006 and 2016 and identified a 9-fold increase in all metabolic surgeries performed in ESKD patients over this time period, largely driven by a profound increase in VSG use (144). Figure 3 outlines the potential roles of metabolic surgery in facilitating access to kidney transplantation and improving allograft outcomes in people with obesity and ESKD.

Table 2 provides an overview of studies which have assessed the impact of metabolic surgery on transplant candidacy in people with advanced non-dialysis dependent CKD or ESKD (145–156). Most studies were small, single-centre retrospective reviews, and without control groups. Study follow-up was generally short (<5 years). VSG was the most studied metabolic surgery type, reflecting its surge in popularity over the past decade (43). BMI <35 kg/m^2^, a threshold below which patients are waitlisted for kidney transplantation at many centres (134), was achieved in >50% of people in all studies in which this outcome was reported. Nevertheless, Cohen et al.'s report of 43 people is the largest observational study of pre-kidney transplant metabolic surgery to date (147). Compared with non-metabolic surgery controls with obesity, pre-transplant metabolic surgery reduced the risks of graft failure and overall mortality by almost 70% (adjusted hazard ratio 0.31 [95% CI 0.29–0.33]) and 45% (adjusted hazard ratio 0.57 [95% CI 0.53–0.61]), respectively (147). Evidence to date supports a growing role for metabolic surgery in addressing obesity as a barrier to kidney transplantation in people with ESKD, and this practice may also improve renal allograft outcomes, although larger studies with longer prospective observational follow-up are needed.

**Table 2:** Observational studies assessing metabolic surgery in people with advanced CKD or ESKD (prior to kidney transplantation).

**Study**	**Year**	***n***	**Surgery type (*n*)**	**Control arm**	**Follow-up (years)**	**T2D [*n* (%)]**	**NDD- CKD [*n* (%)]**	**ESKD [*n* (%)]**	**Dialysis modality (*****n*****)**		**BMI (kg/m**^****2****^**)**		**BMI <35 kg/m^**2**^ [*n* (%)]**	**KTx (*n*)**	**Deceased KTx (*n*)**	**Living KTx (*n*)**	**Mortality (*n*)**
									**HD**	**PD**	**Baseline**	**Last F/u**					
**Kim et al**. **(****145****)**	2019	41	VSG	No	1.8 ± 1.35	23 (56)	2 (5)	39 (95)	–	–	41 ± 5	32 ± 5	–	41	25	16	0
**Yemini et al**. **(****146****)**	2019	24	VSG (17) RYGB (7)	No	3.9 ± 0.5	16 (67)	7 (29)	17 (71)	16	1	42 ± 1	29 ± 1	–	16	5	11	2
**Cohen et al**. **(****147****)**	2019	43	RYGB (27) VSG (9) AGB (6) Unspecified (1)	Yes	3.6 (median)	19 (44)	34 (79)	9 (21)	8	1	43 (median)	32 (median)	–	43	–	–	3
**Thomas et al**. **(****148****)**	2018	31	RYGB	Yes	8 (total)^a^	17 (55)	1 (3)	30 (97)	25	5	44 ± 1	28 ± 1	27 (87)	14	13	1	–
**Kim et al**. **(****149****)**	2017	20	VSG	Yes	3.3	12 (60)	0	20 (100)	–	–	42 ± 4	34 ± 5	–	20	14	6	0
**Kienzl-Wagner et al**. **(****150****)**	2017	8	VSG	No	3.2 ± 1.4	4 (50)	0 (0)	8 (100)	–	–	39 ± 4	31 ± 6	8 (100)	7	7	0	0
**Carandina et al**. **(****151****)**	2017	9	VSG	No	1.3	6 (67)	0 (0)	9 (100)	9	0	46	36	6 (67)	1	–	–	0
**Al-Bahri et al**. **(****152****)**	2017	16	RYGB (12) AGB (3) VSG (1)	No	4 ± 3	–	0 (0)	16 (100)	16	0	48 ± 8	31 ± 7	12 (75)	4	3	1	2
**Freeman et al**. **(****153****)**	2015	52	VSG	No	0.6 ± 0.4	28 (53)	5 (10)	47 (90)	47	0	43 ± 5	36 ± 5	29 (56)	6	3	3	3
**Jamal et al**. **(****154****)**	2015	21	RYGB (18) VSG (2) AGB (1)	No	2.3 ± 1.9	14 (67)	0 (0)	21 (100)	–	–	47 ± 6	35.3 ± 8	–	2	–	–	1
**Lin et al**. **(****155****)**	2013	6	VSG (6)	No	0.5–4^b^	–	1 (17)	5 (83)	5	0	–	–	–	2	–	–	–
**Alexander et al**. **(****156****)**	2007	41	RYGB (41)	No	13 (total)^a^	22 (54)	–	–	–	–	48	–	–	9	–	–	6

a *Total study duration reported where mean follow-up not reported*.

b *Range of follow-up reported where mean follow-up not reported*.

*Mean ± SD are presented for normally distributed continuous variables; n (%) are presented for categorical variables*.

*AGB, adjustable gastric banding; BMI, body-mass index; ESKD, end-stage kidney disease; F/u, follow-up; HD, haemodialysis; KTx, kidney transplant; NDD-CKD, non-dialysis-dependent chronic kidney disease; PD, peritoneal dialysis; RYGB, Roux-en-Y gastric bypass; T2D, type 2 diabetes mellitus; VSG, vertical sleeve gastrectomy*.

The optimal choice of pre-transplant metabolic surgery type in people with ESKD is unknown. VSG is now the most commonly performed metabolic surgery and may be preferable for people with ESKD given the complexities of immunosuppressive medication management in people post-RYGB (mycophenolate mofetil is primarily absorbed in the stomach; the duodenum is the main site of absorption and metabolism of both tacrolimus and sirolimus) (157). Indeed, Thomas et al. demonstrated that people who had RYGB performed pre-transplant required higher tacrolimus doses to achieve comparable trough levels compared with controls with obesity, and were at higher risk of biopsy-proven acute rejection in the post-transplant period (148). The higher biopsy-proven acute rejection rate in RYGB-operated people associated with subtherapeutic tacrolimus trough levels (<4 ng/mL), although several RYGB-operated people were also non-compliant with immunosuppressive medications (148). Similarly, Cohen et al. demonstrated that pre-transplant metabolic surgery (*n* = 38 total, >50% RYGB) increased the risk of acute rejection within the first-year post-transplant by almost 20% compared with non-metabolic surgery controls with obesity (147). As discussed above, urinary oxalate excretion increases post-RYGB with potential for increased stone events and enteric oxalate nephropathy in the renal allograft (158), another factor which has driven the use of VSG in people with ESKD. AGB is a less studied and likely a less favourable surgical option in people with ESKD as the implanted foreign body may increase intra-abdominal infection risks post-transplantation (159). Head-to-head studies between RYGB and VSG in people with ESKD, and between surgical and medical obesity treatment options, would be ideal. Pharmacokinetic studies of immunosuppressive agents post-metabolic surgery are limited (157); larger studies are required, along with clarification of whether acute rejection rates are higher post-RYGB compared with other forms of metabolic surgery.

#### Does the Feasibility of Metabolic Surgery Differ With Dialysis Modality?

From Table 2, it is clear that very few people on peritoneal dialysis have been included in studies of pre-transplant metabolic surgery in ESKD to date. Many studies conducted to date have not outlined the dialysis modalities of included patients, although it is assumed that most included patients have been on haemodialysis where not specifically stated. Surgical interventions pose unique challenges in people on peritoneal dialysis, with potential for surgical damage of the peritoneum requiring interruption of peritoneal dialysis and temporary switching to haemodialysis (with the possibility of haemodialysis catheter complications), dialysate fluid leaks, and peritonitis (160). Conversely, peritoneal thickening and adhesions as a result of long-term dialysis may increase surgical complexity and operative morbidity (160).

The rate of peritoneal dialysis failure as a result of surgery increased as a consequence of open laparotomies (161, 162). Reports of laparoscopic surgical interventions in people on peritoneal dialysis remain limited, although laparoscopic approaches hold promise as a means of maximally preserving peritoneal structural integrity (160). Metabolic surgery procedures are now routinely performed laparoscopically in the general population (39). Valle et al. were the first to report a case series of pre-transplant laparoscopic metabolic surgery in 5 people on peritoneal dialysis in 2012 (163). Imam et al. reported a case of laparoscopic VSG in a 27-year old male on peritoneal dialysis for 3.6 years which did not require perioperative interruption of peritoneal dialysis (164). No perioperative surgical complications occurred and the patient was able to resume his pre-operative dialysis regimen after 4 weeks. Thomas et al. included five people on peritoneal dialysis in their study of pre-transplant RYGB (148), while Yemini et al. and Cohen et al. each included one person on peritoneal dialysis in their studies of pre-transplant metabolic surgery (146, 147). Nevertheless, fewer than 20 cases of pre-transplant metabolic surgery in people on peritoneal dialysis have been reported to date. Further study of the feasibility and efficacy of laparoscopic metabolic surgery is required in this population, particularly to determine if operative morbidity and dialysis complication rates are acceptable and comparable to people on haemodialysis (165).

### Should Metabolic Surgery Be Used to Expand the Living Kidney Donor Pool?

Kidney allograft survival is superior for living compared with deceased donor kidneys (166). Metabolic surgery may increase the living kidney donor pool by addressing donor obesity. As a consequence of the obesity pandemic, >25% of contemporary living kidney donors are obese, compared with <8% in the 1970s (167), and living kidney donation rates have declined by 13% in the United States since 2004 (168). KDIGO recommend that approval of people with BMI ≥30 kg/m^2^ for living kidney donation should be individualized on a case-by case basis and based on the transplant centre's acceptable risk threshold due to higher rates of *de novo* diabetes, hypertension, and ESKD post-donation compared with non-obese donors (169). Additionally, delayed graft function is more common after donation from people with obesity (170). Locke et al. investigated long-term mortality rates in nearly 120,000 living kidney donors with obesity in the United States over 10.7-year median follow-up (171). Obesity increased the relative risk of 20-year mortality by 30% compared with non-obese donors, and although absolute risk of post-donation mortality was low, these findings justify the caution exerted by transplant centres in limiting kidney donation by obese individuals.

Metabolic surgery could potentially improve both donor and recipient outcomes in prospective, obese living kidney donors. Kidney donation is an altruistic act which offers no medical benefit to the donor. However, addressing obesity identified during the donor evaluation process could add tangible medical benefit to donors and potentially reduce post-donation diabetes, hypertension, ESKD, and mortality (57, 58). Nguyen et al. recently provided proof-of-principle that pre-donation metabolic surgery is safe and feasible (172). They performed a case series of 22 people who underwent metabolic surgery prior to living kidney donation across 2 centres in the United States (8 RYGB, 7 VSG, 6 AGB, and 1 BPD/DS). Importantly, none of the metabolic surgeries were performed specifically in preparation for living kidney donation but rather purely for obesity treatment in the donors. Mean pre-operative BMI was 46 kg/m^2^ and 17 donors achieved a post-operative BMI <35 kg/m^2^. Prior metabolic surgery did not impact the donor nephrectomy procedure or early post-donation outcomes. Longer-term donor outcomes and recipient outcomes were not reported.

Thus, it appears that metabolic surgery could be used to expand the living kidney donor pool and potentially improve both donor and recipient outcomes after living kidney donation (Figure 3). Conceivably, this could have even wider downstream impact on paired kidney exchange programmes to circumvent issues of blood group and human leucocyte antigen incompatibility (173). However, many questions remain. Long-term donor and recipient outcomes after pre-donation metabolic surgery have not been characterized (50). The preferred metabolic surgery procedure in this setting is unknown and would ideally be defined through prospective, head-to head studies. Increased kidney stone and oxalate nephropathy risk after RYGB are pertinent to both the donor and recipient in this setting, although the superior metabolic outcomes with RYGB could improve net renal outcomes compared with VSG overall (29, 158).

Metabolic surgery could be performed before, concurrently with, or after kidney donation, and the optimal timing is unknown (174). It seems logical to favour pre-donation metabolic surgery so that benefits of the surgery could also extend to the recipient. Pre-donation metabolic surgery could improve recipient outcomes by reducing glomerular hyperfiltration and other adverse renal consequences of obesity in the transplanted kidney (126). It would also ensure that the prospective donor had adequately responded in terms of weight loss and/or remission of obesity complications such as hypertension prior to proceeding with donation (28). However, laparoscopic metabolic surgery performed concurrently with donor nephrectomy could minimise operative morbidity and recovery time for the donor. An acceptable level of obesity-related comorbidities will need to be defined prior to proceeding with metabolic surgery for prospective living kidney donors (174). While metabolic surgery effectively treats both hypertension and type 2 diabetes (28, 29), given the high rates of type 2 diabetes recurrence at 5 years, it would seem logical to exclude people with type 2 diabetes from studies of peri-donation metabolic surgery for now.

### Can Metabolic Surgery Assist in the Management of Post-Kidney Transplantation Obesity and Metabolic Complications?

Treatment of obesity, hypertension, dyslipidaemia, and diabetes to prevent cardiovascular disease is challenging post-kidney transplantation, partly due to the metabolic side effects of commonly used immunosuppressant drugs. Both cyclosporine and tacrolimus cause hypertension, dyslipidaemia, and pancreatic β-cell toxicity; hypertension and dyslipidaemia are more common with cyclosporine while dysglycaemia and new-onset diabetes after transplantation (NODAT) are more commonly induced by tacrolimus (175). Although steroid-free regimens are sometimes used in allografts of lower immunologic risk, many patients still take a maintenance dose of low-dose glucocorticoid which exacerbates dyslipidaemia and dysglycaemia, and contributes to post-transplant weight gain (176, 177). Mammalian target of rapamycin inhibitors, everolimus and sirolimus, cause dyslipidaemia and may also be toxic to pancreatic β-cells (178). Accordingly, cardiovascular disease remains a leading cause of death post-kidney transplantation, and diabetes is the strongest modifiable risk factor in this setting (179, 180). Furthermore, diabetes is a common cause of renal functional decline in the allograft; over mean 5.4-year follow-up, diabetes accounted for 11% of recurrent and *de novo* glomerular diseases in a large Renal Allograft Disease Registry cohort (181). Death with a functioning allograft remains the leading cause of allograft loss (182). Therefore, better control of conventional cardiovascular risk factors through directed treatment of obesity post-kidney transplantation with metabolic surgery has the potential to improve graft survival and overall patient mortality.

Chen et al. reported a case in which VSG at 3 years post-kidney transplantation significantly improved body weight and glycaemic control in a patient with post-transplant weight gain and NODAT (183). Several small case series and observational studies have investigated the impact of post-transplant metabolic surgery on body weight, metabolic control, and graft outcomes (184–188). Viscido et al. demonstrated that VSG at mean 15 years after kidney transplantation reduced mean BMI from 42.2±6.8 to 29.8±7.3 kg/m^2^ over 1.4±1-year follow-up in five patients (189). Concordant improvements in blood pressure and glycaemic control, lipid parameters, and proteinuria were observed in the majority of patients.

Cohen et al. reported renal allograft outcomes over extended follow-up for patients who underwent post-transplantation metabolic surgery (*n* = 21), and compared graft outcomes for *n*=18 patients to non-metabolic surgery registry controls (*n* = 180) (147). Metabolic surgery (48% VSG, 28% RYGB, 19% AGB, and 5% vertical banded gastroplasty) resulted in 71% excess weight loss at 2 years. Body weight loss did not differ when compared with a cohort of patients who had metabolic surgery performed prior to kidney transplantation. Over median 11.6-year follow-up, *n* = 7 patients experienced allograft failure (consistent with the national average over that time period). Importantly, compared with controls matched for BMI at the time of transplant, metabolic surgery reduced allograft failure by 15% (adjusted hazard ratio 0.85 [95% 0.85–0.86]) and overall mortality by 20% (adjusted hazard ratio 0.80 [0.79–0.82]).

Prospective studies examining the relative impact of metabolic surgery and medical management of obesity on metabolic parameters, allograft function, and mortality should be conducted to further define the role of intentional weight loss strategies in this setting. Caveats pertaining to RYGB highlighted above, including concerns regarding immunosuppressant medication absorption and enteric oxalate nephropathy resulting in allograft loss, may make VSG the metabolic surgery type of choice post-kidney transplant (157, 158). However, adequate control of enteric hyperoxaluria is feasible with appropriate dietary education and oral calcium and citrate supplementation, which may allow a greater number of patients to benefit from the superior body weight and metabolic control achieved with RYGB (29, 92). Additionally, RYGB with a shorter alimentary limb (~75 cm) has the potential to achieve superior metabolic control to VSG while minimising hyperoxaluria and improving immunosuppressive absorption (42), although this requires prospective study in the pre- and post-transplant setting. For people with BMIs close to the limit set by their transplant centre for waitlisting, the optimal timing of metabolic surgery, pre- or post-kidney transplantation, needs to be defined. Pre-transplant metabolic surgery may be preferable as it minimises the early adverse consequences of obesity on the allograft and reduces the incidence of NODAT and other adverse post-transplant metabolic consequences (145).

### Future Directions

Small observational studies support the role of both medical and surgical obesity treatments in people with ESKD, although the longer-term impact of both strategies on kidney transplant waitlisting, kidney allograft outcomes, and overall mortality are unknown. It is unknown whether different metabolic surgery procedures result in differential success in kidney transplant waitlisting and in allograft function. Efforts to recruit more patients on peritoneal dialysis to studies of pre-transplant metabolic surgery should be made, as the majority of studies to date have focused on subjects on haemodialysis. RCTs comparing the impact of medical vs. surgical weight management strategies in people with ESKD would be ideal. Similar to native CKD, combined medical and surgical treatment approaches to obesity may also be set to become the new standard for care for people with ESKD (68). Given the unrelenting obesity pandemic which has increased ESKD prevalence, obesity will become a barrier to kidney transplantation for an increasing number of patients worldwide (35). Therefore, clear guidance on optimal weight management strategies in this setting is critical. Metabolic surgery could help to expand the living kidney donor pool and improve both donor and recipient outcomes, although the optimal timing of metabolic surgery as well as optimal procedure type need to be defined (174). Post-transplant metabolic surgery appears to improve allograft and overall survival (147), and may be increasingly performed to improve cardiovascular risk in those with suboptimal metabolic control, although studies to date have been small with limited longitudinal follow-up.

## Conclusions

There are myriad potential roles for metabolic surgery as a means of improving outcomes for people with kidney disease. Metabolic surgery as an adjunct to medical therapy to slow the progression of diabetic kidney disease currently has the best evidence base and is closest to incorporation into clinical practice. The precise role of metabolic surgery in the diabetic kidney disease treatment algorithm should be defined through randomised controlled trials in people with type 2 diabetes and progressive renal functional decline despite treatment with RAAS inhibitors, SGLT2is, and/or GLP1RAs (8, 9, 190). Many of the mechanisms supporting metabolic surgery use in people with type 2 diabetes and CKD also apply to people with non-diabetic CKD (18); thus, metabolic surgery may assume a broader role in the control of hypertension and proteinuria in non-immune-mediated CKD.

Pre-transplant metabolic surgery improves access to kidney transplantation and improves renal allograft outcomes, decreasing allograft failure and overall mortality (147). Post-transplantation metabolic surgery also improves graft and overall survival (147). Concerns exist regarding the role of RYGB pre- and post-kidney transplantation, particularly with regard to oxalate nephropathy and increased rejection episodes due to unreliable immunosuppressant medication absorption as causes of allograft loss (157, 158). Nevertheless, weight loss and metabolic control are superior after RYGB compared with VSG (29), which may confer incremental renoprotection to the renal allograft. Shortening the length of the alimentary limb should be investigated as a means to optimise renal outcomes post-RYGB by minimising hyperoxaluria and acute rejection risks while preserving superior metabolic control to VSG (41). Finally, metabolic surgery may confer tangible medical benefit to kidney donors by addressing obesity before, concurrently with, or after kidney donation (172).

## Author Contributions

WM and JW wrote the manuscript with critical input from FL-H, ND, and CR. All authors reviewed and approved the final manuscript and as such are accountable for the content of the work.

## Conflict of Interest

CR discloses personal fees outside of the submitted work from Novo Nordisk, GI Dynamics, Eli Lilly, Johnson and Johnson, Sanofi, Aventis, Astra Zeneca, Janssen, Bristol-Myers Squibb and Boehringer-Ingelheim. The remaining authors declare that the research was conducted in the absence of any commercial or financial relationships that could be construed as a potential conflict of interest. The handling Editor declared a past co-authorship with the author CR.

## Abbreviations

AGB: adjustable gastric banding
BMI: body-mass index
BPD/DS: biliopancreatic diversion/duodenal switch
BSA: body surface area
CI: confidence interval
CKD: chronic kidney disease
CKD-EPI: Chronic Kidney Disease-Epidemiology Collaboration
DKD: diabetic kidney disease
eGFR: estimated glomerular filtration rate
ESKD: end-stage kidney disease
FSGS: focal segmental glomerulosclerosis
GLP1RA: glucagon-like peptide-1 receptor analogue
GORD: gastro-oesophageal reflux disease
KDIGO: Kidney Disease Improving Global Outcomes
mGFR: measured glomerular filtration rate
NODAT: new-onset diabetes after transplantation
ORG: obesity-related glomerulopathy
PRFT: peri-renal fat thickness
PUFT: para-renal and peri-renal ultrasonographic fat thickness
RAAS: renin-angiotensin-aldosterone system
RCT: randomised controlled trial
RSF: renal sinus fat
RSNS: renal sympathetic nervous system
RYGB: Roux-en-Y gastric bypass
SGLT2i: sodium-glucose co-transporter-2 inhibitor
SOS: Swedish Obese Subjects
uACR: urinary albumin-to-creatinine ratio
VAT: visceral adipose tissue
VSG: vertical sleeve gastrectomy.
